# Estimating the waiting time of multi-priority emergency patients with downstream blocking

**DOI:** 10.1007/s10729-013-9241-3

**Published:** 2013-05-21

**Authors:** Di Lin, Jonathan Patrick, Fabrice Labeau

**Affiliations:** 1 Department of Electrical and Computer Engineering, McGill University, McConnell, 633 3480 University Street, Montreal, Quebec Canada; 2 Telfer School of Management, University of Ottawa, DMS 7151, 55 Laurier Avenue East, Ottawa, ON K1N 6N5 Canada

**Keywords:** Emergency department, Waiting time, Queueing theory, Hospital management

## Abstract

To characterize the coupling effect between patient flow to access the emergency department (ED) and that to access the inpatient unit (IU), we develop a model with two connected queues: one upstream queue for the patient flow to access the ED and one downstream queue for the patient flow to access the IU. Building on this patient flow model, we employ queueing theory to estimate the average waiting time across patients. Using priority specific wait time targets, we further estimate the necessary number of ED and IU resources. Finally, we investigate how an alternative way of accessing ED (Fast Track) impacts the average waiting time of patients as well as the necessary number of ED/IU resources. This model as well as the analysis on patient flow can help the designer or manager of a hospital make decisions on the allocation of ED/IU resources in a hospital.

## Introduction

Overcrowding in the emergency department (ED) is a worldwide problem [1–3] impairing the ability of hospitals to offer emergency care within a reasonable time frame [4]. By observing more than 20 million patient visits to emergency departments over five years, Guttmann et al. in [5] determined that the risk of death and hospital readmission increases with the degree of crowding in the emergency department, and estimated that about 150 fewer patients would die in Ontario each year if the average waiting time to access the emergency department was less than an hour.

The issue of what constitutes timely access to emergency care is obviously dependent on the acuity of the patient. Without an accurate triage and acuity scale, patients who need immediate emergency care will experience a delay in treatment that may aggravate their condition. The Canadian government published its own acuity guidelines in 1998, and subsequently revised them in 2004 and in 2008. In these guidelines, the severity of patients is classified into five levels: resuscitation, emergent, urgent, less urgent and non urgent [6]. This classification is based on a patient’s presenting complaints, vital signs (including the hemodynamic stability, hypertension, temperature, level of consciousness, respiratory distress, etc.), pain severity, and injury level. For patients in each severity level, their target waiting time to see a physician is detailed in Table 1.

**Table 1:** Triage levels for emergency department [9]

Triage level	Expected waiting time to see a physician
I: Resuscitation	Immediate
II: Emergent	<15 min
III: Urgent	<30 min
IV: Less urgent	<60 min
V: Non urgent	<120 min

However, strict adherence to the priority system may mean that a low priority patient may wait a long time to receive a relatively simple procedure that would tie up ED resources for very little time whereas the higher acuity patients have complex needs that require significant resources. As a means of addressing this issue, many hospitals have introduced a fast-track system for the lower (less urgent and non-urgent) priority patients on the premise that they can be served quickly and easily without tying up too many resources. This policy essentially means that a single queue is broken into two and the ones who inevitably suffer from such a policy are the patients at the end of the first queue (priority III patients). Thus, though the impact of the fast-track system is to reduce over-crowding by serving low acuity patients quickly, it is our contention that, unless resources are increased, this is accomplished at the cost of increasing the wait times for priority III patients. This contention has been partially confirmed. Cooke et al. in [7] and Miquel et al. in [8] show that the fast track can reduce the waiting time for patients who are qualified to access the fast track but will slightly lengthen the waiting time of the other patients. In this paper, we therefore concentrate on a standard priority system without a fast-track but also provide some results for a system with a fast track.

**Fig. 1:**
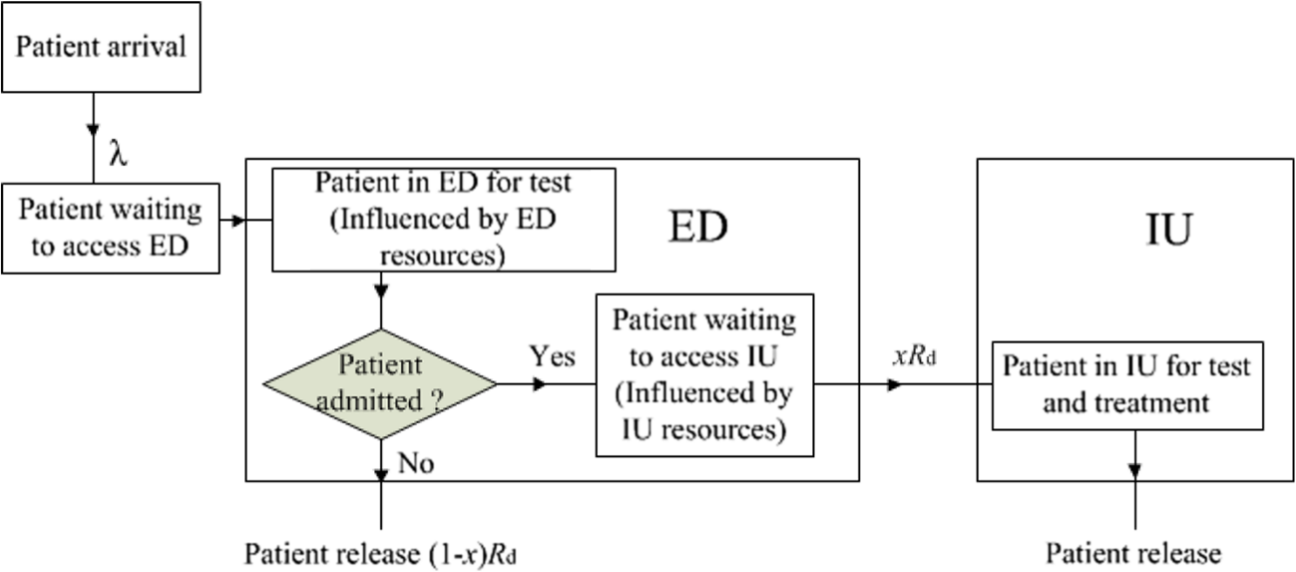
Patient flow to access and depart ED [1]

Even with a good acuity scale in place, insufficient resources can still cause overcrowding. As shown in Fig. 1, the resources available in the ED and in the inpatient unit (IU) will influence patient flow potentially leading to long wait times to access the ED. While the lack of ED resources can block patients in the waiting room, an insufficient number of IU resources may block transfers to the IU further delaying other patients in the waiting room from accessing the ED. Limited budgets however mean that it is inefficient to carry too many resources either in the ED or the IU. Thus, it is imperative that a methodology be developed that correctly estimates the necessary resource capacity in a hospital in order to provide timely access to the ED both to avoid overcrowding and idle time. In this paper, we concentrate on bed capacity in the ED and the IUs. Clearly there are other resources that play a role in determining the service time in the ED such as lab capacity and testing equipment. The assumption here is that other resources are not the rate limiting step to meeting the targets set out in Table 1.

In this paper, we develop a queueing model to estimate the waiting time of patients to access the ED as well as the necessary amount of resources to achieve the wait time targets for each priority class. This queueing model is composed of two queues: the first one is a $M/G/c_{1}/\infty $ with five priorities. In the above notation, the *M* refers to the assumption that the arrival of demand follows a Poisson distribution, the *G* means that the service rate can follow a general distribution and the $c_{1}$ refers to the number of available resources in the ED. This queue characterizes the patient flow from accessing the ED to departing the ED. The five priorities represent five severity levels classified by Table 1. The second queue is a $G/GI/c_{2}/c_{2}$ (general arrival distribution, general but independent service times with $c_{2}$ servers and $c_{2}$ capacity in IU) without priority and no buffer, and it characterizes the patient flow from accessing the IU to discharge.

Additionally, in order to represent the coupling effect that occurs when patients are blocked from entering the IU, we estimate the probability of all servers being busy in the second queue and the probability of patients in the ED waiting for transfer to the IU being blocked. Building on this queueing model, we attempt to estimate the necessary number of ED and IU resources to achieve the performance targets of Canadian Triage And Acuity Scale (CTAS), shown in Table 1.

## Related work

Studies relevant to the estimation of the necessary amount of ED resources can be classified into two stages: (1) Rough estimation stage and (2) further adjustment stage. First, the ED manager needs to estimate the average resource requirements over an extended period of time. Studies in this stream usually estimate the number of resources by the steady state of their estimation models providing a long run average estimation of the resource requirements. This would constitute the first stage. Afterwards, the ED manager may need to adjust the resource assignment to meet the daily fluctuations in demand that are characteristic of ED departments [10–12].

From the perspective of methodologies, while the studies of the first type often utilize queueing [2, 10–12] and Markov chain models [3] to estimate the necessary amount of ED resources in the steady state, the studies of the second type usually utilize an autoregressive integrated moving average model (ARIMA) [13–15], Monte-Carlo simulation [1, 17], or Markov decision process (MDP) [16, 19, 20] to dynamically analyze the necessary amount of resource as a function of time. From the perspective of the length of the planning horizon, the adjustment stage studies can further be classified into short-term estimation and long-term estimation. For short-term estimation (the length of the planning horizon is at most one day) the variation of arrival rate is measured at the hourly level [13–16]. For long-term estimation (the length of observation ranges from more than one day to a few months), the daily variation or even seasonal variation is factored into the planning decisions [19, 20].

Our study is of the first type, namely, estimating the steady state resource requirements in a hospital in order to provide timely access to the ED. To our knowledge no paper has dealt with both the coupling effect between two resources (such as the ED and IU) as well as the presence of multiple priority classes in the demand stream. Patrick et al. in [19, 20] present how to estimate the required capacity for a diagnostic imaging department dealing with multiple priority classes but deal with only a single resource. Kolb et al. in [1, 17] take into account the coupling effect between the ED and the IU but do not consider the different triage levels of patients. Similarly, Koizumi et al. in [21] present a model that deals with a series of resources with potential blocking but with no acuity scale differentiating patients. For the purposes of estimating resource requirements in the ED to achieve priority-specific performance targets, it is important to propose a model that takes into account both factors. The model proposed in the next sections achieves that goal.

## Models of patient flow

In this section, we present the arrival process of emergency patients as well as the model to describe this process. Building on this model, we estimate the average waiting time of an emergency patient accessing the ED.

Patients arrive at the ED either as walk-in patients or ambulatory patients. After entering the main entrance, the patient moves from the greeter desk to triage to registration before waiting for a physician consult. In our model these steps are combined under the heading ’Patients waiting to access the ED’ (see Fig. 1). Once a patient accesses the ED, they are assigned a bed by a nurse. After a pre-examination by the ED nurse, the patient will see a physician for further examination, tests and treatments. If the physician determines the patient can be discharged after examination, an ED nurse facilitates the process of releasing the patient who then departs the ED. If the physician decides to transfer the patient to the inpatient units (IUs), a consulting physician from the IU will arrange the admission. Depending on the availability of IU beds, the patient is either transferred immediately or has to wait in the ED till an IU bed becomes available. These steps in our model are combined under the heading ’Patients staying in the ED’. A patient in the IU will receive tests and treatments until he/she is deemed ready for discharge. After a final examination by a physician, the patient will depart the IU. These steps in our model are combined under the heading ’Patients staying in the IU’.

### Arrival process of demand accessing the ED and the IU

Demand for ED service fluctuates significantly throughout the day. In consequence, Koizumi et al. in [21] divide the whole day into several segments such that the arrival rate in each segment can be assumed to be constant. They demonstrate that the arrival process of emergency patients in most time segments can be modeled as a Poisson process with varying rates.

As mentioned in the previous section, our model looks to provide the steady state resource requirements over a longer period than a day and thus we do not take into account the within day variations. Given the results from our model, a further model (perhaps using a Markov Decision Process approach) could be used to allocate the available resources in order to take into account the within-day fluctuations in demand.

The data presented in Table 2 was provided by a local hospital and represents the average arrival rate of patients entering the ED per hour (broken down by CTAS) and the average arrival of patients entering the IU per hour (broken down by point of entry) for fiscal year 2011/2012.

**Table 2:** Patient flow into the ED and the IU

Destination	CTAS/Point of Entry	Arrival Rates
ED	I	0.075
	II	0.662
	III	3.749
	IV	2.86
	V	0.226
	All	7.572
IU	From ED	0.479
	Direct	0.267
	All	0.746

### Queueing models to estimate the waiting time

Building on the flowchart of patients accessing and departing the ED (shown in Fig. 1), we use queueing theory to estimate the waiting time of a patient to access the ED with the waiting time of a patient referring to the time between arriving at the waiting room until treatment. To estimate the waiting time, we need to model two patient flows. (1) The patient flow from arriving at the waiting room until departing the ED can be viewed as a $M/GI/c_{1}/\infty $ queue to model the patient flow arriving at and departing from waiting rooms or ambulances since the size of the waiting room is rarely the limiting factor. A $M/GI/c_{1}/\infty $ queue has a Poisson arrival process, independent and identically distributed service times with a general distribution, $c_{1}$ ED beds and infinite buffer capacity. (2) The patient flow into the IU through to discharge can be viewed as a $G/GI/c_{2}/c_{2}$ queue, which has a general arrival process, independent and identically distributed service times with a general distribution, $c_{2}$ IU beds and no buffer capacity.

#### Model for patient flow in the ED

Let $\lambda $ represent the arrival rate of emergency patients and $\mu _{1}$ represent the average service rate in the ED without taking into account the coupling effect. Due to the potential for patients being blocked from leaving the ED, we can adjust the average service time in the ED, $1/\mu $, (represented by a $M/G/c_{1}/\infty $ queue) as

1 $$ 1/\mu=1/\mu_{1}+P_{b}\times mean(\mathop {min}\limits_{i} T_{i}) $$

where $T_{i}~(i=1,2 \cdots c_{2})$ is the waiting time until *i*th inpatient is discharged and $P_{b}$ is the blocking probability to access the IUs. The rate of patients transferring to the IU is $x R_{d}$, where $R_{d}=min\{\lambda , c_{1} \mu \}$ and *x* is the proportion of patients transferred into the IU.

Thus, this modified average service time represents the actual service in the ED plus the time spent waiting in the ED for an IU bed. The time waiting to access the IU equals the average length of waiting until the next inpatient departure, denoted as $\mathop {min}\limits _{i} T_{i}$.

To incorporate the priority classification system of emergency patients, we use the theory for a preemptive resume multi-priority $M/G/c_{1}/\infty $ queue to calculate the length of waiting time to access the ED. The rationale for using preemptive resume queue models is that high priority (Level I or level II) patients must receive immediate service. If no physician is available when such a patient arrives, a physician treating a lower priority patient (or stabilized higher priority one) must leave his/her current patient and resume only after they offer the emergency treatment to the high priority patient. The detailed calculation on the length of the waiting time is presented in Section 4.

#### Model for patient flow in the IU

The flow of patients accessing and departing the IUs can be modeled as a $G/GI/c_{2}/c_{2}$ queue [22]. In this queueing model, we focus on the blocking probability $P_{b}$, that is, the probability that there are no available beds in the IUs. This can be determined by

2 $$ P_{b} = \frac{\alpha \beta e^{{ - k\beta } \mathord{\left/{\vphantom {{ - k\beta } v}} \right. \kern-\nulldelimiterspace}v}}{(1 - e^{{ - k\beta } \mathord{\left/ {\vphantom {{ - k\beta } v}} \right. \kern-\nulldelimiterspace} v}){\rho_{d}} \sqrt {c_{2}}} $$

where $c_{2}$ is the number of beds available in the IUs, $\lambda _{d}$ is the arrival rate of patients directly accessing the IUs and $\mu $ is the service rate. Other parameters in Eq. 2 can be calculated as:

$$\begin{array}{rll} \rho _{d} & = \frac{xR_{d} + \lambda _{d} }{c_{2} \mu}, \quad & \beta = \sqrt {c_{2}} (1 - \rho_{d}), \\ k & = \sqrt{c_{2}}, \quad & v = \frac{1 + C_{a}^{2} }{2}, \\ \alpha & = \left[ {1 + {\beta \Phi (\beta )} \mathord{\left/ {\vphantom {{\beta \Phi (\beta )} {\varphi (\beta )}}} \right. \kern-\nulldelimiterspace} {\varphi (\beta )}} \right]^{ - 1}, \end{array} $$

where $C_{a}^{2}$ is the squared coefficient of variation (SCV) of the service time at inpatient units and $\Phi ( \cdot )$ and $\varphi ( \cdot )$ are the cumulative distribution function (CDF) and probability density function (PDF) of a standard normal distribution.

### Steady state conditions of queuing models

In this paper, we employ queueing models to represent the steady state of a queueing system to access the ED. As shown in Fig. 1, the patient flow for accessing and departing the ED is represented by two coupled queueing models. In the following, we will explore the conditions for both queueing models to reach the steady state.

Given the arrival rate $\lambda $, the necessary and sufficient steady state condition for the first queue is $\lambda \le c_{1} \mu $, and that for the second queue is $xR_{d} + \lambda _{d} \le c_{2} \mu _{I}$. Given the parameters of these two queues, we can decide whether these queues can reach steady state if we know the exact $\mu $, which determines both the service rate in the first queue and the arrival rate of the second queue. Unfortunately $\mu $ is interdependent with another unknown parameter $P_{b}$ (shown in Eqs. 1 and 2), and neither of them can be shown in a closed form. Thus, the key to attaining the necessary and sufficient steady state conditions for both queues is to calculate $\mu $. In the following, relaxing the constraints, we first present a sufficient (not necessary) condition as well as a necessary (not sufficient) condition without requiring the exact $\mu $. Second, we develop an iterative algorithm to numerically compute $\mu $ from which a necessary and sufficient steady state condition can be approximated.

#### *Sufficient (but not necessary) and necessary (but not sufficient) conditions*

As the blocking probability satisfies $0 \le P_{b} \le 1$, we can obtain constraints on $\mu $ as $\mu _{min} \le \mu \le \mu _{max}$, where

3 $$\mu_{min}  =1 \mathord{\left/ {\vphantom {1 {(1 \mathord{\left/{\vphantom {1 {\mu _{1} }}} \right. \kern-\nulldelimiterspace} {\mu _{1}} + mean(\mathop {min}\limits_{i} T_{i} ))}}} \right. \kern-\nulldelimiterspace} {(1 \mathord{\left/ {\vphantom {1 {\mu _{1}}}} \right. \kern-\nulldelimiterspace} {\mu _{1} } + mean(\mathop{min}\limits_{i} T_{i} ))} \text{ and }$$

4 $$\mu_{max}  =\mu _{1},  $$

where $\mu _{min}$ is the value of $\mu $ (refer to Eq. 1) when $P_{b}=1$ (the worst case in which an ED patient transferring into IU is always blocked), and $\mu _{max}$ is the value of $\mu $ when $P_{b}=0$ (the best case in which an ED patient transferring into IU is never blocked). Thus, the sufficient steady state condition of the first queue is $\lambda \le c_{1} \mu _{\min }$, and its necessary steady state condition is $\lambda \le c_{1} \mu _{\max }$. Correspondingly, the sufficient steady state condition of the second queue is $x\min \{\lambda ,c_{1} \mu _{\min } \} + \lambda _{d} \le c_{2} \mu _{I}$, and its necessary steady state condition is $x\min \{\lambda ,c_{1} \mu _{\max } \} + \lambda _{d} \le c_{2} \mu _{I}$.

#### *Necessary and sufficient condition*

In the following, we will discuss the necessary and sufficient steady state conditon. First of all, we represent Eq. 1 as

5 $$ y_{1}(\mu)+y_{2}(\mu)-1/\mu=y_{0} $$

where $ y_{1}(\mu )=1/\mu $, $y_{2}(\mu )=1/\mu -P_{b} \times mean(\mathop {min}\limits _{i} T_{i})$, and $y_{0}=1/\mu _{1}$. In the following, we show that there is at least one feasible solution to Eq. 5, and this solution $\hat {\mu } \in [\mu _{min}, \mu _{max}]$.

#### Proof

We will prove it by contradiction. Let $y(\mu )=y_{1}(\mu )+y_{2}(\mu )-1/\mu $. From Eq. 2, we know $y_{2}(\mu ) \le y(\mu ) \le y_{1}(\mu )$. Assume that there is no feasible solution to Eq. 5, then $y_{0}=y_{2}(\mu ) < y(\mu )$ at $\mu =\mu _{min}$, otherwise $\mu _{min}$ will be the feasible solution. Because of the continuity of $y(\mu )$ and our assumption of no feasible solution in $[\mu _{min}, \mu _{max}]$, we can deduce that $y(\mu )>y_{0}$ for $\mu \in [\mu _{min}, \mu _{max}]$. At $\mu =\mu _{max}$, we can conclude that $y(\mu )>y_{0}=y_{1}(\mu )$, which is contradicted by the fact $y(\mu ) \le y_{1}(\mu )$ from Eq. 2. □

**Fig. 2:**
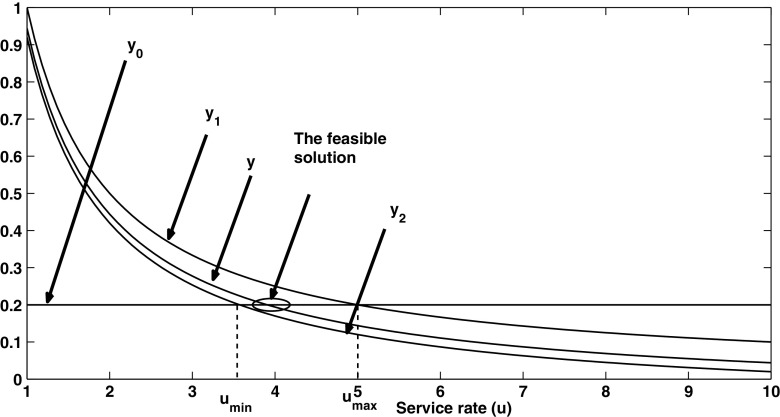
Intuitively understanding the proof

Beyond the proof, we can also demonstrate that at least one solution exists intuitively from Fig. 2. Certainly a feasible solution exists (shown in circle), because of the constraints: $y_{2}(\mu ) \le y(\mu ) \le y_{1}(\mu )$, $y_{2}(\mu _{min})=y_{0}$, and $y_{1}(\mu _{max})=y_{0}$.

If there is only a single solution $\hat {\mu }$, then it is easy to show the necessary and sufficient steady state condition as $\lambda \le c_{1} \hat {\mu }$ for the first queue and $x\min \{\lambda ,c_{1} \hat {\mu }\} + \lambda _{d} \le c_{2} \mu _{I}$ for the second queue.If there are multiple solutions, then no necessary and sufficient steady state condition exists. In the latter case, we can at least find a tighter sufficient condition and a tighter necessary condition by replacing $\mu _{min}$ and $\mu _{max}$ by $\hat {\mu }_{min}$ and $\hat {\mu }_{max}$, respectively. Please note that $\hat {\mu }_{min}$ and $\hat {\mu }_{max}$ are the minimal and maximal feasible solutions. The detailed process of searching for the minimal and maximal feasible solutions is shown in Algorithm 1. 
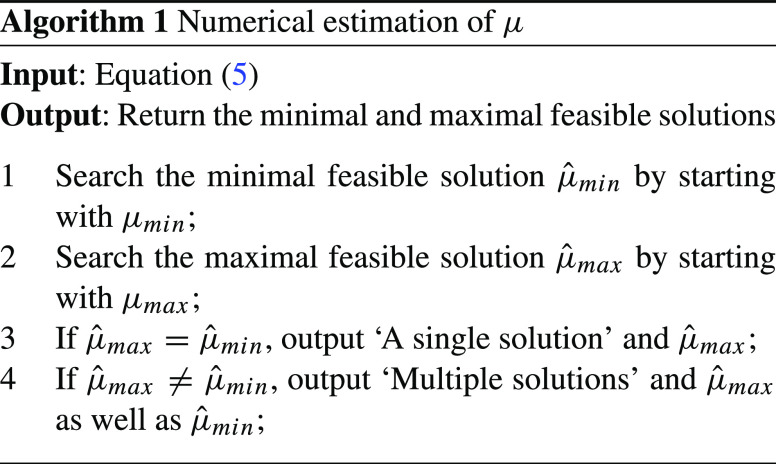


If $\hat {\mu }_{min}$ and $\hat {\mu }_{max}$ 1 are the same, the sufficient and necessary condition exists and this condition is: $\lambda \le c_{1} \hat {\mu }$ for the first queue and $x\min \{\lambda ,c_{1} \hat {\mu }\} + \lambda _{d} \le c_{2} \mu _{I}$ for the second queue. Otherwise, there is no sufficient and necessary condition, and we can use the tightest sufficient condition: $\lambda \le c_{1} \hat {\mu }_{min}$ for the first queue and $x\min \{\lambda ,c_{1} \hat {\mu }_{min}\} + \lambda _{d} \le c_{2} \mu _{I}$ for the second queue to guarantee the stability of the queue.

## Estimation of the waiting time using the queueing models

In this section, we present the estimation of the waiting time in the ED. As shown in Eq. 1, this waiting time is dependent on $mean(\mathop {min}\limits _{i} T_{i})$, namely, the average waiting time to be transferred to the IUs. In the following, we first estimate $mean(\mathop {min}\limits _{i} T_{i})$ and then estimate the waiting time in the ED. Also we take into account two cases: patient flow with a fast track and without a fast track. Specifically, we present the estimation of waiting time in the case of no fast track in IV. B., and the case with a fast track in IV. C.

### Estimation of the waiting time to be transferred into the IUs

In this section, we discuss how to calculate the average waiting time to access the IUs, namely, $mean(\mathop {min}\limits _{i} T_{i})$. Given $F_{i}(z)$ as the cumulative distribution function (CDF) of $T_{i}~(i=1,\cdots ,c_{2})$, which is estimated as a triangular distribution in [17], the CDF of $\mathop {min}\limits _{i} T_{i}$ (denoted as $F_{min}(z)$) can be expressed as [18]

6 $$ F_{\min } (z) = 1 - \prod\limits_{i = 1}^{c_{2} } {\left[ {1 - F_{i} (z)} \right]} $$

Building on Eq. 6, we can calculate the mean of $\mathop {min}\limits _{i} T_{i}$ as

7 $$ mean(\mathop {min}\limits_{i} T_{i})=\int {zdF_{\min } (z)} $$

By substituting Eqs. 6 and 7 into Eq. 1, we can attain the expression of the modified average service time $1/\mu $.

### Estimation of the waiting time in the ED

As presented in Table 2, emergency care patients can be classified into 5 categories. Patients with severity level I and II are provided priority over ambulatory patients waiting for admittance to the ER. The prioritizing frequently includes pre-empting or suspending service for patients fitting the profile of categories IV and V so that appropriate care can be provided in life threatening situations [29]. From the perspective of queuing models, a pre-empting multiple-priority queue is appropriate for this scenario [28–30].

As presented in III.B.(1), we will use a $M/G/c/\infty $ queue to characterize the patient flow. Few studies provide the exact closed-form expression of waiting time in a $M/G/c/\infty $ queue. The exact waiting time is difficult to calculate because such a queue does not possess an embedded Markov chain [24]. Instead, most studies focus on an approximation of a $M/G/c/\infty $ queue. Hokstad in [25] and Miyazawa in [26] both suggest using a probability generating function (PGF) to generate an approximation of $M/G/c/\infty $ queues. Tijms et al. in [27] propose a regenerative approach to simulate a $M/G/c/\infty $ queue. The aforementioned approximation methods are reliable but all require knowledge of the exact distribution of the service time, which is not applicable in our scenario because the distribution of our modified service time is unknown (shown in Eq. 1).

By using heuristics, Bondi et al. in [31] present the estimation of the waiting time in a preemptive resume multi-priority $M/G/c_{1}/\infty $ queue. Given the number of priorities *K*, the mean waiting time of patients with priority $k~(k=1,\cdots ,K)$, $W_{c_{1} }^{k}$, can be estimated as [31]

8 $$ W_{c_{1} }^{k} \approx \frac{W_{c_{1} }^{F} W_{1}^{k} }{W_{1}^{F} } $$

where $W_{1}^{k}$ represents the mean waiting time of patients with priority *k* in a preemptive resume multi-priority $M/G/1/\infty $ queue, $W_{1}^{F}$ represents the waiting time of a FCFS $M/G/1/\infty $ queue and $W_{c_{1}}^{F}$ represents the waiting time of a First-come, first-served (FCFS) $M/G/c_{1}/\infty $ queue. Here we note that the priority increases with *k* such that priority *k* represents the triage level $K+1-k$, shown in Table 1. In the following, we focus on computing $W_{1}^{F}$, $W_{c_{1}}^{F}$, as well as $W_{1}^{k}$.

#### Estimation of $W_{1}^{F}$

The waiting time in a FCFS $M/G/1/\infty $ queue can be easily computed following [32]

9 $$ W_{1}^{F} = \frac{\lambda S^{2}}{2(1 - \rho )} $$

where $\rho =\lambda S$, $\lambda $ represents the arrival rate of patients across all priorities, *S* represents the mean service time for patients across all priorities and $S^{2}$ represents the second moment of the service time of patients across all priorities.

#### Estimation of $W_{c_{1}}^{F}$

The waiting time in a FCFS $M/G/c_{1}/\infty $ queue has been estimated in [33] as

10 $$ W_{c_{1} }^{F} \approx \left[ {S + \frac{SP_{Q} }{c_{1} - \lambda S}} \right]\frac{1 + C^{2}}{2} $$

where $C^{2}$ represents the SCV of the service time for patients across all priorities, $\lambda $ represents the arrival rate of patients across all priorities and *S* represents the mean service time for patients across all priorities. $P_{Q}$ represents the probability given in [33] as

11 $$ P_{Q} = \frac{\left( {c_{1} \rho } \right)^{c_{1} }}{c_{1} !(1 - \rho )}\left[ {\sum\nolimits_{t = 1}^{c_{1} - 1} {\frac{\left( {c_{1} \rho } \right)^{t}}{t!}} + \sum\nolimits_{t = c_{1} }^{\infty} {\frac{\left( {c_{1} \rho } \right)^{t}}{c_{1} !c_{i}^{t - c_{1} } }} } \right]^{ - 1} $$

where $\rho = {\lambda S} \mathord {\left / {\vphantom {{\lambda S} {c_{1} }}} \right . \kern -\nulldelimiterspace } {c_{1} }$.

#### Estimation of $W_{1}^{k}$

The waiting time in a preemptive resume multi-priority $M/G/1/\infty $ queue has been well studied. Specifically, given the number of priorities *K*, for each priority $k=1,\cdots ,K$, the mean waiting time is expressed in [32] as

12 $$W_{1}^{k} =\left\{ \begin{array}{ll}  \frac{(1 - \rho _{1} )S_{1} + R_{1} }{1 - \rho _{1} }, & \hbox{for $k=1$} \\  \frac{(1 - \rho _{1} - \cdots - \rho _{k} )S_{k} + R_{k} }{(1 - \rho _{1} -\cdots - \rho _{k - 1} )(1 - \rho _{1} - \cdots - \rho _{k} )}, & \hbox{for $k>1$} \end{array}\right.$$

where $\rho _{k}=\lambda _{k} \overline S _{(k)}$, $\lambda _{k}$ represents the arrival rate of patients with priority *k*, $\overline S _{(k)}$ represents the mean service time for patients with priority *k* (after taking into account the coupling effects), $R_{k} = \frac {1}{2}\sum \nolimits _{i = 1}^{k} {\lambda _{i} \overline S _{(k)}^{2} }$, and $\overline S _{(k)}^{2}$ is the second moment of the service time of *k*th-priority patients.

#### Estimating the mean and second moment of service time in the ED

Building on Eqs. 10, 11 and 12, the estimation of $W_{1}^{F}$, $W_{c_{1}}^{F}$, and $W_{1}^{k}$ are determined by the mean and second moment of service time for patients in each priority as well as the average across various priorities. Building on Eq. 1, we can attain the mean and second moment for *k*th-priority patients as in Eq. 13 below.

13 $$ \overline S _{(k)} = S_{(k)} + P_{b} [mean(\mathop {min}\limits_{i} T_{i} )] $$

and

$$\begin{array}{rll} \overline S _{(k)}^{2} &=& S_{(k)}^{2} + 2S_{(k)} P_{b} \times mean(\mathop {min}\limits_{i} T_{i} ) \\ &&+ P_{b}^{2} [mean(\mathop {min}\limits_{i} T_{i} )]^{2} \end{array} $$

where $S_{(k)}$ represents the emergency service time in the ED, and $S_{(k)}^{2}$ represents the second moment of emergency service time in the ED.

The average service time of the first queue across various priorities, $\mu $, equals the average of the mean service time of each priority. Namely, $\mu =1 \mathord {\left / {\vphantom {1{\sum \limits _{k} { q_{k} \overline S _{(k)} } }}} \right . \kern -\nulldelimiterspace } {\sum \limits _{k} { q_{k} \overline S _{(k)} } }$, where $q_{k}=\lambda _{k}/\sum \limits _{k}{\lambda _{k}}$. Substituting this into Eq. 2, we can denote $P_{b}$ as a function of $\sum \limits _{k} {\overline S _{(k)} }$.

To compute the mean and the second moment of service time for patients in each priority, we develop a numerical algorithm, shown in Algorithm 2. The Algorithm 2 iteratively calculates $S _{(k)}$, $S _{(k)}^{2}$, $P_{b}$, and $\mu $ until the estimation of $S _{(k)}$ and $S _{(k)}^{2}$ in two consecutive iterations has a difference below a threshold. If the output is not ’No solution’, then, this output can be viewed as an estimation of the exact mean and second moment of the service time within an acceptable difference. Building on this algorithm as well as Eqs. 10, 11 and 12, we can attain the estimation of the waiting time of patients to access the ED, $W_{c_{1}}^{k}$. 
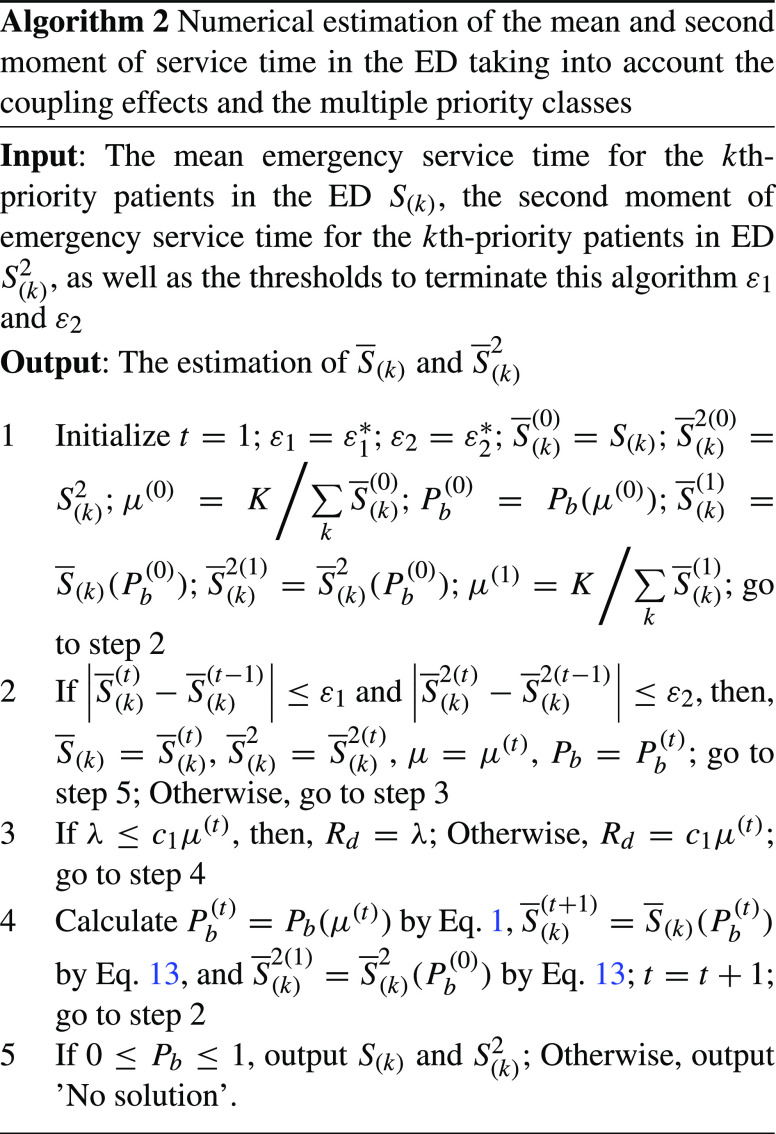


### Estimation of waiting time for an ED with a fast track

While the previous sections investigated the case of ED without a fast track, we now turn our attention to the case with a fast track. A fast track is designed for patients with less serious illnesses and injuries to shorten both waiting and treatment times for these patients. More specifically, after evaluation by a triage nurse, patients with less emergent issues (triage level IV and V) are placed in the fast track [34].

In our model, a system with a fast track places paitents at triage level IV and V on an express line, and the other patients will be put on the regular line. Also $c_{1}$ beds in ED will be split into two parts: one part for Fast Track (the number of beds is denoted as $c_{1}^{I}$) and one part for regular line (the number of beds is denoted as $c_{1}^{II}$). According to Eqs. (1) and (2), the averge service time (denoted as $1/\mu ^{I}$) in the Fast Track and the average service time (denoted as $1/\mu ^{II}$) in the regular line are shown as Eq. 14.

14 $$\begin{array}{rll} 1/\mu^{I} &=& 1/\mu_{1}^{I}+P_{b}\times mean(\mathop {min}\limits_{i} T_{i})\\ 1/\mu^{II} &=& 1/\mu_{1}^{II}+P_{b}\times mean(\mathop {min}\limits_{i} T_{i})\\ \mu_{1}^{I}+\mu_{1}^{II} &=& \mu_{1} \end{array} $$

where $P_{b}$ is shown in Eq. 2.

The average service time (denoted as $1/\mu ^{F}$) across all triage levels is shown in Eq. 15

15 $$\begin{array}{rll} &&{} 1/\mu^{F}=\frac{1}{P_{r}^{I} \mu^{I}+ P_{r}^{II} \mu^{II}}\\&&{} P_{r}^{I}+P_{r}^{II}=1 \end{array} $$

where $P_{r}^{I}$ is the probability of a patient whose triage level is at level I-III, while $P_{r}^{II}$ is the probability of a patient whose triage level is at level IV–V.

By substituting Eq. 15 into Eq. 8, we can estimate the average waiting time of Fast Track by setting the number of beds in ED as $c_{1}^{I}$, while estimating the average waiting time in the regular line (acute side) by setting the number of beds in ED as $c_{1}^{II}$. Definitely, both of these waiting times are dependent on the proportion of patients who are switched over to the fast track, namely, the probability of a patient whose triage level is at level I–III $P_{r}^{I}$.

## Result and discussion

In the following we investigate the necessary capacity of ED and IU in various scenarios to meet the waiting time targets from arrival to first physician assessment in the ED (shown in Table 1). For triage level I, we replace ’immediate’ by ’$<$3 min’. The other parameters in our model include the arrival rate $\lambda $ as the average arrival rate of emergency patients throughout the day (shown in Table 2) and the arrival rate $\lambda '$ as the average arrival rate of patients that directly access the inpatient units throughout the day (shown in Table 2). In addition, the distribution of the service time in the ED is triangular with a lower limit of 0.1 hour, upper limit of 1 hour, and mode of 0.5 hour (see Section 4.1). The distribution of the service time in the IU is triangular with a lower limit of 1 day, upper limit of 7 days, and mode of 4 days. Building on Eq. 8, we can estimate the waiting time to access the ED without a fast track as well as with a fast track, and compare the results in both scenarios in Section 5.4. The other parameters in Eqs. 1–10 can be calculated from the aforementioned parameters.

### Relationship between ED and IU resource requirements

**Fig. 3:**
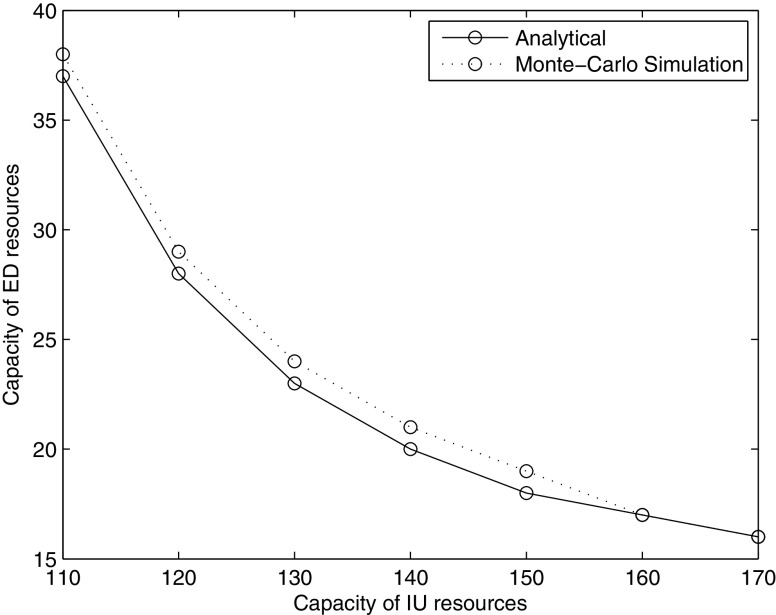
Capacity of ED resources vs. capacity of IU resources

Given a set of priority specific wait time targets, the model presented here can be used to estimate the necessary capacity both in the ED and the IU in order to meet the performance targets. Figure 3 shows the impact of changes in the available IU capacity on the necessary ED capacity. In our queueing model, the average of length of stay in the IU is 4 days, the arrival rate to access the ED is 7.572 patients per hour, and we assume here that there is no fast track. Also our analytical results are verified by Monte-Carlo simulations, in which we mimic two individual queues which link with each other and calculate the necessary capacity of ED resources by repeating the simulation 50000 times.

Unsurprisingly, the necessary ED capacity increases as the size of the IU decreases. However, the impact of additional IU capacity on ED resource requirements decreases significantly as the IU increases suggesting that there is a threshold size of the IU beyond which additional increases cease to be advisable. However, attempting to reduce the size of the IU below that threshold leads to a steep increase in the required ED capacity in order to meet the same targets and therefore is likely not cost-effective.

### Capacity of resources and length of stay in the IU

**Fig. 4:**
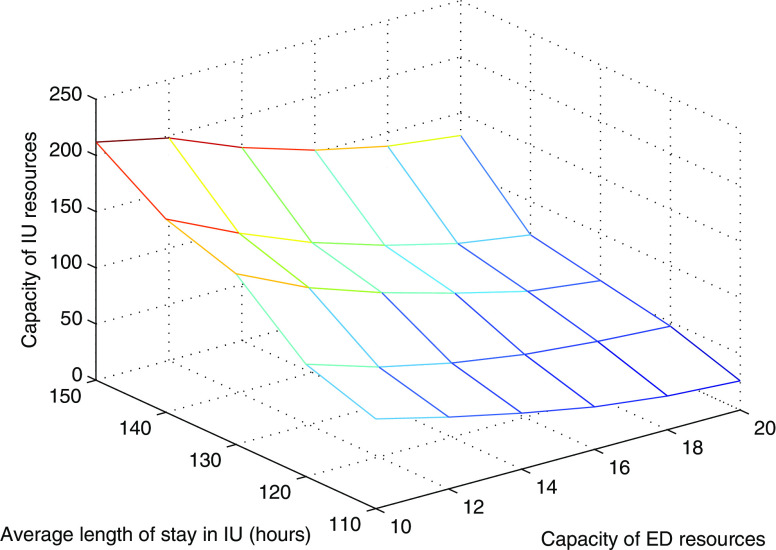
Capacity of ED/IU resources vs. average length of stay in IU

Clearly, the probability of congestion in the IU leading to backlogs in the ED is dependent on the rate of turnover in the IU which is regulated by the length of stay of patients. Thus, it is of interest to analyze changes in the necessary capacity of the IU/ED resources as the length of stay in the IU is varied. In the queueing model, we first vary the average length of stay in IU through 110–150 hours. Given a number of ED resources, we can determine the necessary IU resources as the length of stay in the IU is varied. Secondly, by varying the number of ED resources through 10–20, we can obtain the 3-dimensional plot which determines how the necessary ED/IU resources change as the length of stay in the IU varies (shown in Fig. 4). In both cases, we fix the arrival rate to the ED at 7.572 patients per hour and assume that there is no fast track.

With the increase in the length of stay in the IU, the capacity of both IU resources and ED resources will, of course, increase. However, the rate of increase in the necessary IU resources as the length of stay is increased is significantly higher than for the ED (shown in Fig. 4). In other words, an increase in the length of stay in the IU causes a larger increase in the necessary IU resources. Thus, in the face of greater uncertainty in the length of stay of patients, it is preferable to carry additional or “excess” IU capacity rather than hoping to manage this problem by using the ED as a holding bay for the IU; a reality that is surprisingly common in practice.

### Capacity of resources and arrival rate of ED patients

Obviously, the necessary ED and IU resources to guarantee an acceptable waiting time to see a physician are dependent on the arrival rate to the ED, so it is of interest to investigate the impact on ED and IU resource requirements of increases in this arrival rate. We focus on how much additional ED/IU capacity should be anticipated as necessary given a forecast of increasing demand. In the queueing model, we first fix the number of IU resources at 125 and determine the necessary ED resources as the arrival rate to the ED is varied. Secondly, we fix the number of ED resources at 20 and determine how the necessary IU resources change as the arrival rate to the ED is varied. In both cases, we fix the length of stay in IU at 150 hours and assume that there is no fast track.

**Fig. 5:**
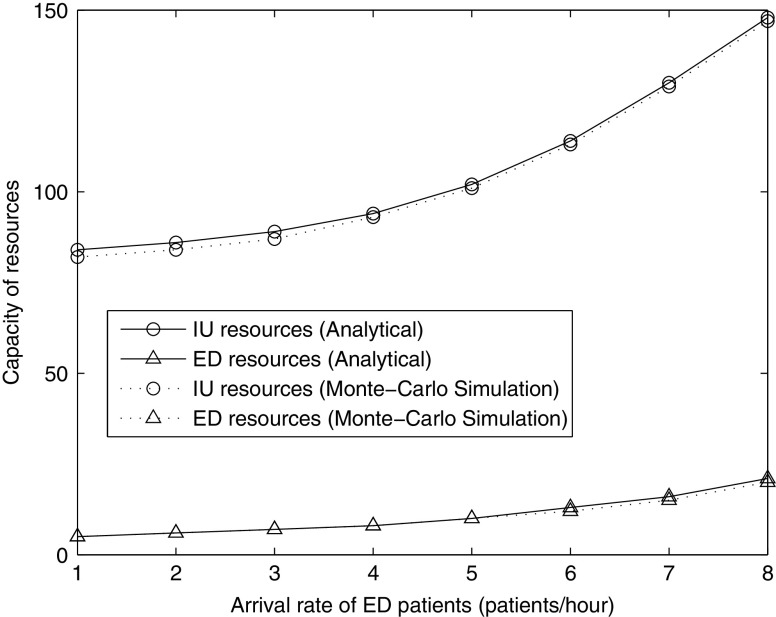
Capacity of ED/IU resources vs. arrival rate of ED patients

With the increase in the arrival rate to the ED, the required capacity of ED and IU resources will, of course, increase. However, the rate of increase in the necessary IU resources as the arrival rate is increased is significantly higher than for the ED (shown in Fig. 5). In other words, an increase in the arrival rate to the ED causes a larger increase in the necessary IU resources. Thus, again in the face of greater uncertainty in the arrival rate of ED patients, it is preferable to carry additional or “excess” IU capacity rather than hoping to manage this problem by using the ED as a holding bay for the IU.

### Average waiting time to access the ED with and without a fast track

The aforementioned discussion assumes that a hospital does not implement a fast track for patients at triage level IV and V. In the following, we focus on the influence of a fast track on the average waiting time to access the ED. In this scenario, the average length of stay in the IU is 4 days, and the number of IU resources is 125. In the scenario with a fast track, we allocated 20 % of ED capacity to the fast track.

**Fig. 6:**
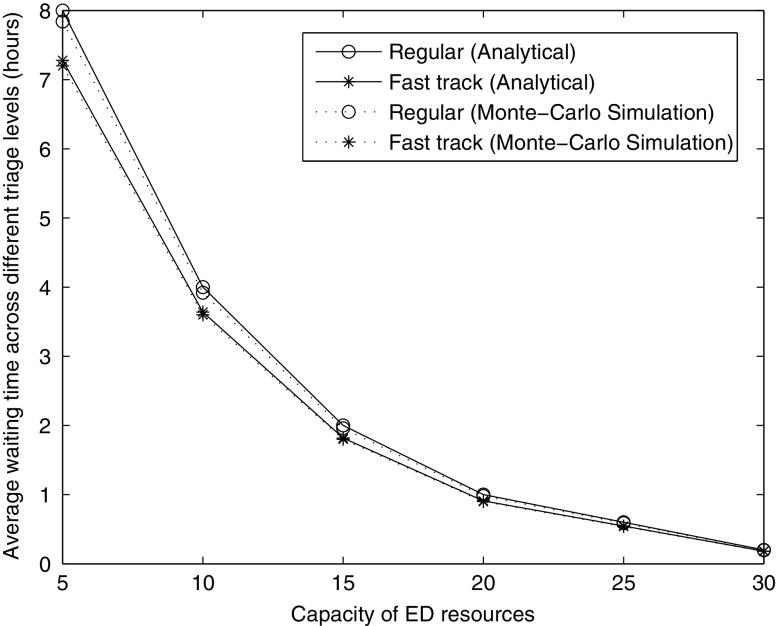
Average waiting time across various triage levels vs. capaciy of ED resources

As shown in Fig. 6, a fast track reduces the average waiting time across all patients to access the ED by approximately 0.3–1 hour. The explanation is as follows. The fast track provides for the prompt treatment of patients with non-life-threatening injuries. In a non-fast track system, these patients are forced to queue behind patients whose complexity requires long service times. The fast track provides short wait times for a cohort of patients who otherwise would have the longest wait, thus reducing the average wait time.

**Fig. 7:**
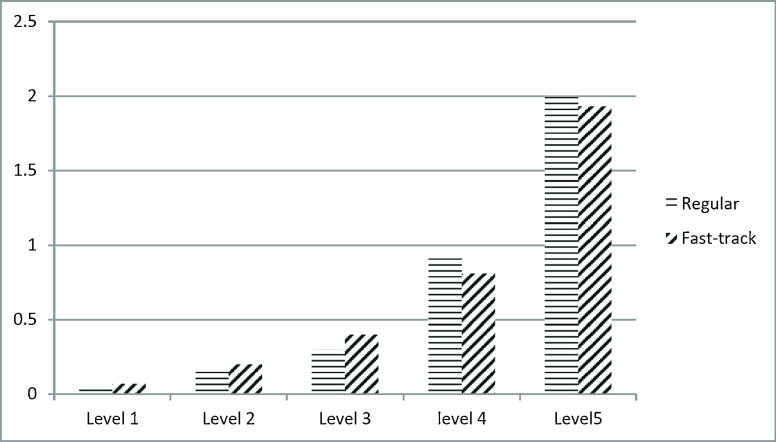
Waiting time to see a doctor across various triage levels [hours]

**Fig. 8:**
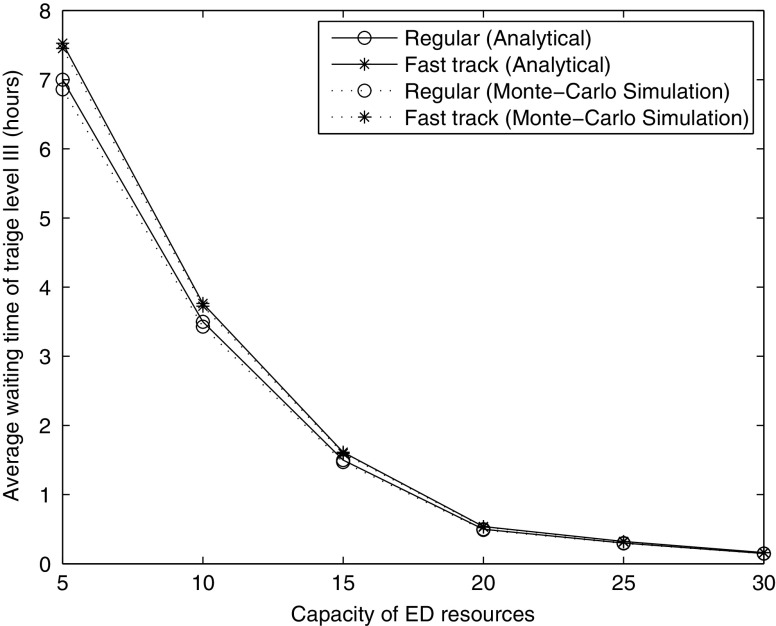
Average waiting time of triage level III vs. capaciy of ED resources

However, the reduction in the average waiting time across all patients comes at the cost of increasing the waiting time of patients who do not qualify for the fast track. As shown in Figs. 7 and 8, the average waiting time of patients at triage level III increases by around 0.1–0.5 hours in a system that implements a fast track. In this scenario, the number of ED resources is 25.

### Limitations of our patient flow model

The patient flow model in our paper has the following limitations: firstly, we assume that the inpatient unit can accommodate all types of patients, but in reality, a few specialized units can only accommodate a specific type of patient. For example, an injured patient cannot stay in a cardiovascular disease unit. Secondly, in our model, we assume that one resource can only be offered to one patient, so *c* patients will consume *c* resources in the ED. However, in real scenarios, EDs with *c* beds can accommodate more than *c* patients by using hall beds and internal waiting rooms [35]. Thirdly, our model employs steady-state and average arrival rates, which do not take into account that the arrival rate is non-stationary. Finally, our model does not cover the real-life cost implications of Length of Stay (LOS) reductions.

## Conclusions

In this paper, we develop a two-stream model to characterize the coupling effect between patient flow to access the ED and that to access the IU. Building on this patient flow model, we employ queueing theory methods to estimate the average waiting time across patients as well as the necessary ED and IU resources in order to meet target waiting times for patients at each triage level. In addition, we investigate the influence of a fast track stream on the average waiting time of patients. Our model improves on previous research by taking into account both the reality of multiple priority classes competing for ED resources and the strong potential for downstream congestion impacting on the timely access of patients to the ED.

In addition to providing hospital management with a means of determining the necessary capacity in the ED and the IU in order to meet priority specific wait time targets for timely access to the ED, this paper also provides the following insights. (1) There is a threshold size for the IU such that reductions in the IU below that threshold lead to steep increases in the necessary ED capacity in order to meet the same targets. Thus, there exists a “optimal” IU capacity such that either increasing or decreasing IU resources will lead to higher costs associated with the same performance. (2) Uncertainty in the LOS in the IU or in the arrival rate to the ED is best accommodated by carrying extra IU capacity rather than by increasing the size of the ED. (3) While a fast track can reduce the average waiting time across patients from all the triage levels, it is accomplished by a large reduction in the wait times of patients from triage levels IV and V that offset a concurrent increase in the wait times of patients from triage level III. In other words, a fast track actually worsens the ability of an ED to provide timely access to patients whose conditions could potentially progress to a more serious problem requiring emergency intervention, such as asthma, vaginal bleeding, moderate trauma, Gastrointestinal (GI) bleeding, and acute pain. These three insights are partially verified by relevant research literature [7, 8, 36]. Erik et al. in [36] explain that increasing IU resources instead of ED resources with the aid of “IU buffers” can solve the problem of overcrowding and shows the “best buffering capacity” in IU, but this paper does not take into account the triage levels of patients. Also Cooke et al. in [7] and Miquel et al. in [8] show that the fast track can reduce the waiting time for patients who are qualified to access ED but will slightly lengthen the waiting time of the other patients. However, these two papers do not consider the coupling effects between the ED and IU.

We would like to extend our results by varying the proportion of ED resources allocated to the fast track, and develop a general rule to determine the optimal allocation depending on the proportion of demand that is eligible for the fast track and the relative resource consumption of the two groups. We would like to explore whether there exists an optimal proportion of ED resources to allocate to the fast track in order to minimize the demand of IU resources and satisfy all CTAS time requirements.

## Footnotes

1 Searching for the closest solution from the starting point can be done by any standard method. We use the Matlab function ‘fsolve’ to carry out the search, and its default internal algorithm is the trust-region dogleg algorithm [23].
